# Psychosocial Support Programme Improves Adherence and Health Systems Experiences for Adolescents on Antiretroviral Therapy in Mpumalanga Province, South Africa

**DOI:** 10.3390/ijerph192315468

**Published:** 2022-11-22

**Authors:** Emeka Francis Okonji, Brian van Wyk, Gail D. Hughes, Ferdinand C. Mukumbang

**Affiliations:** 1 School of Public Health, University of the Western Cape, Cape Town 7535, South Africa; 2 Medical Biosciences Department, University of the Western Cape, Cape Town 7535, South Africa; 3 Department of Global Health, University of Washington, Seattle, WA 98195, USA

**Keywords:** adolescents living with HIV, psychosocial support, HIV, AIDS, adherence, retention

## Abstract

(1) Background: Psychosocial support (PSS) plays a significant role in persistent adherence to and retention in antiretroviral therapy (ART) for adolescents living with the human immunodeficiency virus (ALHIV). This paper qualitatively explores the experiences of ALHIV on ART, who participated in a PSS programme in five public primary healthcare facilities in Mpumalanga Province in South Africa during the COVID-19 pandemic. (2) Methods: Data were collected through 24 focus group discussions with 173 ALHIV on ART and subjected to inductive thematic analysis. Informed consent was obtained before all data collection. (3) Results: The PSS programme facilitated the process of full HIV disclosure to these adolescents with the support of parents/guardians while motivating adherence through peer support groups and health education for improved treatment literacy. Participants reported positive health systems experiences, improved healthcare provider–client relations, and prompt access to health services. (4) Conclusions: The PSS programme successfully kept ALHIV engaged in ART care despite the health service disruptions encountered during the COVID-19 pandemic. We recommend rigorous evaluation of the effects of the PSS intervention on adherence to and retention in ART among ALHIV in HIV-endemic settings.

## 1. Introduction

Adolescents account for 5.9% of the burden of HIV, thus representing the fastest-growing age group of people living with HIV globally [1]. In 2016, an estimated 1.2 million HIV-positive children and adolescents lived in eastern and southern Africa, constituting approximately 90% of those living with HIV in Africa [2]. Despite tremendous gains in reducing AIDS-related deaths by 43%, AIDS-related deaths among adolescents in eastern and southern Africa have increased in the last decade [2]. The reason is that adolescents fail to adhere to treatment and find it challenging to remain engaged in antiretroviral therapy (ART).

The World Health Organization defines adolescence as a period of life between 10 and 19 years [3]. Adolescence is when the child transits into adulthood and moves towards greater independence [3]. Concurrently, the adolescent develops a sense of autonomy and a desire to establish an individual identity [4]. In this period of life, many physiological and emotional changes occur within the individual [5]. Adolescents living with HIV (ALHIV) face complex challenges as they explore their sexual identity and form relationships [6], along with challenges with HIV status disclosure [7] and understanding the importance of effective treatment options while on ART [8]. On the other hand, this period is also characterised by high-risk sexual and other behaviours that could be detrimental to their health and well-being [9]. It is argued that despite the well-known need for HIV prevention and reducing reproductive health risks, their age, social, and financial status often limit adolescent access to information and services in many settings [10].

Mental health issues, including neurodevelopmental and cognitive deficits, are also common among ALHIV because of the chronic nature of the disease, fear of HIV disclosure, stigma and discrimination, and depressive disorders [11,12]. Research indicates that HIV-positive adolescents tend to have high levels of common mental health problems that directly affect adherence to ART and retention in care negatively [13,14]. Thus, the impact of common mental health illnesses on the quality of life, in turn, affects adherence to ART and retention in care [12].

While psychosocial interventions for HIV-affected adults have shown improved adherence to and retention in care [15], the knowledge base for ALHIV is sparse. The few studies reported little to moderate impact of psychosocial support (PSS) on adherence to and retention in care among ALHIV. In this paper, we aim to qualitatively explore the experiences of a PSS intervention delivered to ALHIV in five primary healthcare facilities in the Ehlanzeni District of Mpumalanga, South Africa, during the COVID-19 pandemic.

## 2. Materials and Methods

### 2.1. Study Setting

According to the 2017 National HIV Prevalence, Incidence, Behaviour and Communication survey, the Ehlanzeni District has the second-highest HIV prevalence in South Africa, with an estimated prevalence rate of 17.3%. In our baseline study, we found a 74% viral suppression rate (confidence interval: 73.1–74.8%) among ALHIV on ART in 2019 [16], and retention in care rates at 6, 12, 18, and 24 months of 90.5%, 85.4%, 80.8%, and 76.2%, respectively [17], which remain far from the UNAIDS target of 95–95–95 by 2030. This study was conducted in five public primary healthcare facilities, Buffelspruit Clinic, Kanyamazane Community Health Centre (CHC), Cunningmoore Clinic, Langloop CHC, and Naas CHC.

### 2.2. Overview of the Right to Care Psychosocial Support Intervention

**Figure 1:**
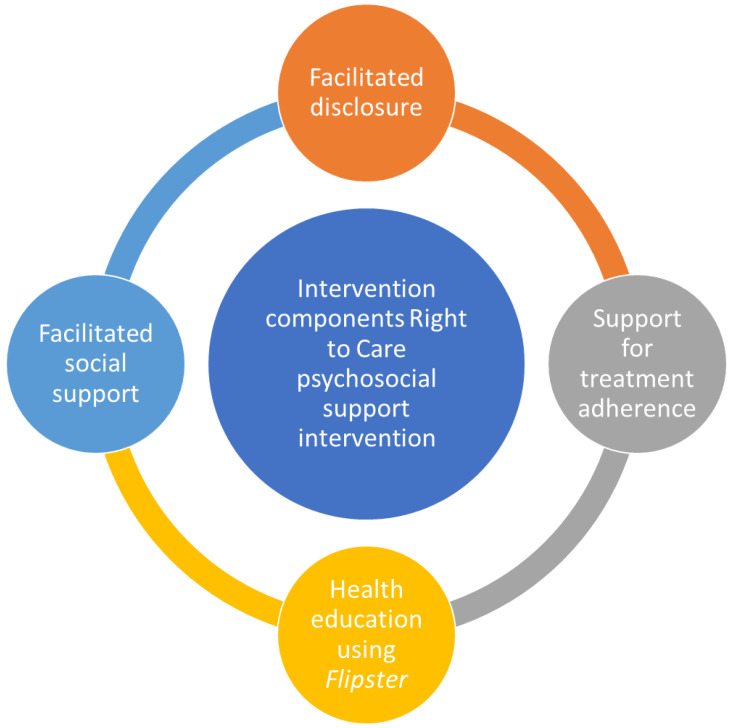
Intervention components of the Right to Care psychosocial support intervention.

Right to Care (RTC) is a registered South African non-governmental organisation that provides quality healthcare services, including prevention, treatment care, and support services for people living with HIV. RTC developed a PSS programme consisting of adolescent and youth-friendly services that aim to improve adherence and retention to ART care among ALHIV. The comprehensive PSS programme package comprises services to address disclosure, treatment adherence, social support, and HIV treatment literacy among adolescents and young adults (ages 10 to 24 years) who are unaware of their HIV-positive status. Adolescents and young adults living with HIV are enrolled on adherence support groups (Figure 1) [18].

The intervention utilises peer supporters to support participants’ adherence to and retention in ART care. The peer supporters work with the healthcare system and obtain the names and addresses of adolescents and young who have tested positive for HIV from local healthcare facilities. With the adolescents’ assent and consent from their parents/caregivers, the peer supporters register them into the Right to Care psychosocial support (PSS) programme. As participants, the adolescents and young adults are guided and counselled on the importance of adherence to and retention in care and are followed up regularly. In most cases, the peer supporters also fast-track the collection of ARVs for the beneficiaries when they visit the health facilities.

Participants are organised into groups by age (10–13, 14–16, and 17–24 years). The support groups aim to empower adolescents and young people living with HIV to become resilient, better informed, and better able to make well-informed choices about managing their HIV status. The peer supporters are trained to use the psychosocial-oriented Flipster facilitation model developed by RTC to facilitate support group sessions with participants at selected ‘safe spaces’.

### 2.3. Study Design, Sampling, and Data Collection

Between the 1 and 30 November 2021, 24 focus group (FGD) discussions were conducted with ALHIV (10–19 years old) on ART who received the PSS programme in five public primary healthcare facilities in Ehlanzeni District. All seven facilities that implemented the PSS programme were included in this study. We applied a qualitative descriptive research design. Participants were purposively selected from five facilities implementing the PSS intervention to obtain a diverse experience of ALHIV receiving the PSS programme. Face-to-face contact was made with participants, their parents/caregiver to brief them on the aim of the research.

FGDs were conducted in the clinics with four groups of ALHIV (*n* = 173); females 10–14 (*n* = 42) and 15–19 years old (*n* = 49); and males 10–14 (*n* = 33) and 15–19 years old (*n* = 49) (Table 1). Those who did not participate gave reasons for being busy on the day of the FGD. ALHIV at Mashinshing and Masibeka clinics did not honour their appointments due to heavy rains and flooding that affected their households. At Buffelspruit Clinic, while only nine ALHIV females (10–14 years old) participated in the FGD, four male ALHIV (10–14 years old) who came for the FGD discontinued because they became emotional and were referred to a counsellor.

FGD guides were developed in English, translated into Tsonga, back-translated into English, revised, and piloted for clarity. FGDs were conducted in Tsonga and facilitated by two researcher assistants who took notes during the discussion. FGDs were audio-recorded, transcribed, and translated into English by the researcher who facilitated that group’s discussion. Local research assistants participated in a week-long training workshop on qualitative data collection techniques and had daily de-briefings with a trained supervisor and ongoing supervision.

### 2.4. Data Analysis

Two researchers coded the data from the FGD, and they were analysed using an iterative process. An initial codebook was developed deductively from the interview guide and inductively from transcripts. The inductive content analysis technique we conducted is suitable for analysing qualitative data collected without the guidance of a theoretical framework but deriving purely from emergent data and formulating themes [19]. Data were captured in an Excel table with five columns in the order of categories, themes, subthemes, codes, and quotations or remarks (Appendix A). We developed codes from the responses provided by the participants. Data codes were reviewed by the authors and later organised into subthemes, themes, and categories (pattern coding). We further read and cross-checked the transcribed data to confirm the developed pattern coding. Data saturation was achieved where no new themes and subthemes emerged from the FGD at a group and individual level.

### 2.5. Trustworthiness and Credibility

The trustworthiness and credibility of the study were ascertained by doing the following. First, we piloted the interview questionnaires with ALHIV to ensure that the questions were culturally relevant and sensitive to solicit a response. Second, we conducted iterative questioning in data collection dialogues with the study participants. Third, to ensure that all ALHIV participated in the discussion, they were assured that there were no wrong answers as their experiences were paramount. Finally, we followed the relevant aspects of the consolidated criteria for reporting qualitative research (COREQ) outlined by Tong et al. [20,21]. The COREQ checklist aimed to promote clear and comprehensive reporting guidelines for qualitative studies.

### 2.6. Ethics Approval and Informed Consent

The researchers sought verbal and written consent from all study participants and parents/caregivers when participants were younger than 18. Pseudonyms were used to identify study participants in the transcription of FGD. In addition, participants consented to the publishing of their responses if their identities were kept anonymous. No personal information was collected from participants during the research process.

## 3. Results

The objectives of the PSS programme were to facilitate and support disclosure, optimise treatment effectiveness, and provide health education and social support to improve adherence to and retention in ART among ALHIV. The themes, subthemes, and codes derived are aligned with these programme objectives and illustrated in Appendix A. A fifth category (recommendations) was formed to capture those themes that did not fit into the initial ones identified but captured “recommendations” to improve the PSS intervention. In this section, we started by capturing the experiences of the ALHIV pre-PSS era and then during the PSS implementation era.

### 3.1. Pre-PSS Programme

The adolescents were asked how they felt after learning about their HIV status (including those who got to know about their HIV status by themselves) before joining the PSS programme. The participants (ALHIV) reported that they felt depressed, hopeless, confused, and angry, became reclusive, blamed their parents, had low self-esteem, and started smoking. In addition, they had poor health/treatment literacy about HIV, which negatively impacted adherence to and retention in ART.

### 3.2. Psychological Distress and Maladaptive Behaviours

After knowing their HIV status and before enrolling on the PSS programme, the typical psychological challenges experienced by ALHIV included poor mental health, social and emotional difficulties, and substance abuse.

#### 3.2.1. Poor Mental Health

ALHIV, who reported challenges with their mental health, reported having depressive symptoms because of frequent crying because of knowing their HIV-positive status. Some of them expressed that they cried a lot after learning about their HIV status:

*I cried* [frequently cried] *and did not want to go to school*. 15-year-old, female Kanyamazane CHC

Additionally, others expressed feelings of hopelessness:

*I felt like dying as I thought it’s over with my life: I was nine years* [old] *when I found out*. 17-year-old female, Langloop CHC

#### 3.2.2. Social Challenges

ALHIV who learned about their HIV status on their own before a formal disclosure no longer wanted to interact with peers or relatives. They blamed their parents for their HIV status.

*I no longer wanted to interact with anyone*. 14-year-old female, Buffelspruit Clinic

Another stated:

*I was angry at my parents, so I did not want to speak*. 17-year-old female, Cunningmoore Clinic

#### 3.2.3. Emotional Challenges

The ALHIV experienced emotional challenges in the form of low self-esteem, feeling confused and angry, and lack of affection from parents and relatives after being disclosed their HIV status.

*I became emotional and had low self-esteem after knowing my status*. 14-year-old male, Naas CHC

*I was furious and upset*. 15-year-old female, Kanyamazane CHC

*I was confused, not knowing what implications that [I] was supposed to have in my daily life*. 13-year-old male, Cunningmoore Clinic

*Back in the day, I was not getting much love [from family] before I even knew my status. It’s not nice for me, and it’s painful.* 18-year-old male, Naas CHC

#### 3.2.4. Substance Abuse

Behavioural challenges in the form of smoking and drinking were common coping strategies used by ALHIV upon learning about their HIV-positive status.

*This* [knowing my HIV-positive status] *changed my life, and I started smoking and drinking (laughing). I even started smoking weed to make me high and come back late from friends going home*. 16-year-old female, Langloop CHC

### 3.3. Health Literacy

Health literacy is people’s knowledge, motivation, and competencies to access, understand, appraise, and apply health information to make judgments and decisions in everyday life. Decisions concern health care, disease prevention, and health promotion to maintain or improve quality of life during the course of life [22]. In the FDG with ALHIV, we found that inadequate health literacy had a negative impact on medication adherence.

*Since it was flu medication, I would skip it since I didn’t know that I am* [was] *to drink* [it] *for a long time*. 14-year-old male, Cunningmoore Clinic

ALHIV who knew their HIV status commonly reported having poor psychological well-being and inadequate health literacy, leading to sub-optimal adherence to and retention in ART care.

### 3.4. Post-Intervention

As part of the post-intervention, we recounted the experiences of the ALHIV attending the PSS programme. Based on our findings, the PSS programme facilitated disclosure of HIV status to the ALHIV. It provided health education and an opportunity for a strengthened social support network to improve adherence to and retention in ART care.

### 3.5. Facilitated Disclosure of HIV Status

Adolescents who were perinatally infected with HIV lacked an understanding of continuous medication intake. Full disclosure was a vital component of the PSS programme in assisting the parents/caregivers to overcome the fear of disclosing to their children. It also clarified the need for adherence and a criterion for recruiting ALHIV into adolescent support groups. The disclosure component of the intervention was facilitated by trained clinicians, providing clarity to ALHIV on their continuous ART intake, enhancing their ability to take responsibility for this, and subsequently improving medication adherence.

The ALHIV narrated how they felt content after disclosure and knowing that they were not alone, also that their positive HIV status could be managed with accurate knowledge and support. One ALHIV stated:

*The white Doctor told me, and I was happy because I finally got an answer about what this medication was for, unlike not knowing*. 14-year-old female, Langloop CHC

Another ALHIV reported:

*…. because on the day they disclosed to me, other children were in the old clinic, and we were told that we were all the same. We all have the virus, and there is nothing to be afraid of. That made me feel happy because there were lots of us that day*. 13-year-old female, Langloop CHC

It was noted that facilitating disclosure and counselling encouraged the ALHIV to accept their HIV status.

*My mother told me it does not start with me, and it does not end with me. I should also accept because it is not her fault, and as time went by, I started coming to the clinic and accepted since I am not the only one and I shall overcome it*. 18-year-old female, Kanyamazane CHC

Furthermore, the ALHIV reported having renewed hope after receiving counselling and knowing that HIV is not an end to life. Additionally, that one can live an ordinary life if one continues to adhere to and remain in ART care.

*We feel like we have our whole life and future ahead of us irrespective of our status*. 15-year-old male, Naas CHC

Disclosure of their HIV status facilitated by a trained clinician and the counselling received from clinicians was observed to improve the psychological well-being of ALHIV, also renewing their expectations for the future.

### 3.6. Health Education

The PSS programme enabled a conducive environment for the ALHIV to discuss crucial topics on HIV treatment literacy and address challenges to medication adherence. We present ALHIV’s responses on how the health education they received, using a *Flipster* training manual, improved their treatment literacy about HIV and the importance of antiretroviral (ARV) drugs, a better understanding of the virus, and improved adherence to and self-management of ART.

*Importance of ARV Medication Adherence*

The health education provided to the adolescents enabled them to understand the importance of ARV medication. One ALHIV narrated:

*I would get sick, and they* [clinicians] *found out that I was not taking my pills, and I was reprimanded for not taking my medications. I was told these pills would help me live … without the ART. I would get sick, lose weight, and end up dead. I continued taking them. I was also told always to eat my food so that by the time I take my pills, I have something in my stomach and should drink a lot of water. So, I took that and continued up until now*. 13-year-old male, Naas CHC

*A Better Understanding of the Virus*

Similarly, the health education provided to the ALHIV gave them a better understanding of the virus. They recounted how their perceptions of the virus improved based on what they had been taught in the PSS programme.

*It is important to take the medication, so you don’t have AIDS. The treatment makes the body strong and improves the immune system*. 13-year-old female, Kanyamazane CHC

*I think it’s fine so that the soldiers of the body can stay strong in our bodies*. 18-year-old male, Kanyamazane CHC

*Improved Adherence*

The health education provided to ALHIV reportedly improved their adherence to ART. According to the ALHIV, they explained what could happen to them if they do not adhere to their medication.

*I was taking my treatment well, but when I got to grade 7, the problem began since I was not taking it well. At times I would take it, and other times I wouldn’t, so I would have a week without taking it, which happened for a month. When I went back to the clinic for my blood, that was when it was explained to my mother and me that I was not taking my treatment, so they explained more what happens when I do not take it, I will die, and from that day, I don’t miss taking my pills*. 16- years-old male, Naas CHC

*Self-Management of ART*

As a result of the health education and counselling received during the PSS programme, the adolescents living with HIV were able to take responsibility for their medication intake. According to an ALHIV, it was noted that:

*I am taking responsibility* [for] *my health, and I remind myself every time, and even my little sister does remind me, and if I don’t take* [it], *she would even report to my mom. So, I thank her a lot she is always there to remind me*. 18-year-old female, Kanyamazane CHC

The health education sessions provided to ALHIV taught them about the importance of ARV, empowered them with a better understanding of HIV, improved adherence to ARV drugs, and aided self-management of ART.

### 3.7. Counselling and Encouragement

During the health education sessions, ALHIV were counselled and encouraged to continue taking ARV drugs and never miss clinic appointments.

*They encourage and tell us every day that we should take care of ourselves and take treatment on time so that we can be healthy kids, and sometimes they take us out for fun walks so that we can relieve stress, and that is very helpful to us as kids*. 14-year-old female, Cunningmoore Clinic

### 3.8. Strengthened Social Support Network

One of the objectives of the PSS programme was to facilitate social support for improved access to ART (medication pick up and clinic attendance) and retention in care by strengthening the ALHIV’s support networks. The PSS programme facilitated peer support, parents/caregivers support, and improved client–clinician relationships and health service delivery.

#### 3.8.1. Peer Support

ALHIV, who attended the support group sessions, expressed the feeling of not being alone and, as such, are motivated to take their medication regularly. Being motivated resulted from their interaction with other children who share similar HIV-positive experiences and face similar challenges, such as fears of stigma and discrimination.

*This support group has helped me a lot. It is no longer like before, and I know that I am not alone and there’s more of us, and I can talk to someone about a challenge I have and ask if they have the same challenge and if so, we can get to share the solutions and remind each other to take treatment. At least now I have someone with experience just like me, and I can share [discuss] everything I encounter, and I am happy*. 18-year-old male, Kanyamazane CHC

*I like the fact that we meet and discuss similar problems. We are open to each other and share similar problems, and it’s nice to know there are others who are going through the same problems*. 13-year-old female, Naas CHC

*I take my treatment regularly because I am attending with others that are taking treatment*. 13-year-old male, Kanyamazane CHC

In addition, the ALHIV expressed improved self-esteem because of the support they received from each other and their parents/caregivers during their treatment journey.

*Now I feel proud of myself. I no longer look down on myself. The support I’m getting from this group and home has significantly helped*. 13-year-old female, Cunningmoore Clinic

#### 3.8.2. Enhanced Parent/Caregiver Support

Support group sessions attended by ALHIV revealed them being supported by their parents/caregivers to take their medication regularly, as these carers acted as their role models. Being reminded and using reminder strategies such as TV programmes and emotional support also played a role. This is because the parents/caregivers were involved during the disclosure process.

*Since both my parents are taking it [ARV drugs],-that made me continue taking it because I know that I am not alone*. 14-year-old male, Buffelspruit Clinic

*She* [mother] *always makes sure that I take my ART regularly; even if she is at work, she calls to check if I have taken my ART*. 13-year-old male, Langloop CHC

*Television also helps. I know that every time ‘Generations’ plays on TV, it automatically reminds me to take my ART*. 13-year-old male, Langloop CHC

*I used to cry a lot and always wanted to be alone and not talk to anyone. I had a difficult time with my anger, but the person who was very supportive in my life was my grandmother. She helped me with the burden and made me understand that this virus was not a death sentence*. 13-year-old female, Buffelspruit Clinic

#### 3.8.3. Client–Clinician Relationship

ALHIV attended the support group sessions, bonding with clinicians and having quick access to treatment. The result was their interaction with friendly healthcare workers who facilitated the support group sessions and pre-packed their ARV medication for pick-up.

*Yes, it does help me because I can ask the nurse a lot of things, and with my peers as well, I can share whatever is bothering me, and if I forget to take my pills, I ask them what they do if they forget or if they don’t. So, it* [is] *beneficial*. 18-year-old female, Buffelspruit Clinic

*She* [professional nurse] *is very open and easy to talk to, and she is not the type of person who gets angry or even shout*[s] *at us. She behaves like us so that she can understand us better*. 11-year-old female, Cunningmoore Clinic

On the experiences of quick access to health services, ALHIV stated that:

*It’s great to meet in groups, we get assisted very fast and arrive home early because you don’t queue*. 12-year-old male, Langloop CHC

*So far, it is working for me because I don’t take time to queue when I am here, and here, I sit for an hour or 30 min*. 15-year-old female, Kanyamazane CHC

The PSS programme provided ALHIV with facilitated support and care from peers, parents/caregivers, and clinicians, as well as prompt access to ART, improving adherence to ART and retention in care.

### 3.9. Recommendations to Improve PSS

ALHIV provided suggestions to improve the PSS programme being implemented. These include reminders to collect medication, psychological motivation, help with transport to the clinic, and confidentiality of clients’ clinical files.

The ALHIV indicated that they would like to receive reminder calls or messages to collect their medication.

*Before, if I am supposed to come on Sunday, they can call me in the morning and remind me*. 13-year-old male, Langloop CHC

ALHIV also suggested they needed a motivational speaker for regular motivation and encouragement.

*We will appreciate* [it] *if we* [ALHIV] *can have counsellors to motivate us maybe once or twice a year so that we can continue to take our ART with confidence because that encourages and give*[s] *us hope*. 15-year-old female, Buffelspruit Clinic

*If we can get a motivational speaker to motivate us about taking our ART, even if it can be twice a year*. 14-year-old female, Naas CHC

The ALHIV suggested transportation to enable them to attend support group meetings and pick up their medications at the clinics, especially those far from the clinic.

*Maybe help us* [ALHIV] *with transport to the facility because we are far*. 14-year-old, female Langloop CHC

ALHIV offered ways that they would like to see clinicians or healthcare workers handle their clinical files in a confidential manner. The ALHIV said they would prefer the clinic to be discreet in handling their clinical files when visiting the clinic.

*Our concern is that when we collect our medication, we have files, while those not on treatment don’t have files. So, we suggest a plan be made for us not to be publicised by files*. 17-year-old female, Cunningmoore Clinic

## 4. Discussion

In this study, we qualitatively explored the experiences of a PSS programme designed to improve adherence and retention of ALHIV on ART in the Ehlanzeni District in Mpumalanga Province, South Africa. The programme components included facilitated disclosure and social support, as well as support for treatment adherence and health education. We found that the PSS programme improved their understanding of HIV and its treatment and enabled them to build stronger bonds with peers, parents/caregivers, and clinicians. However, there is a need for motivational messaging to create renewed hope for the future and provide transport to attend clinic visits.

Disclosure is found to be empowering and essential for improving adherence and medication acceptance, besides increased responsibility for self-management [23]. However, the evidence base for the association between disclosure and adherence is mixed. While some studies found a positive association between disclosure and adherence [24,25,26,27,28], others reported no association [29,30,31,32,33]. Interestingly, some studies found an opposite effect between disclosure and adherence [34,35,36,37], which was linked to depressive symptoms [34] and denial of HIV status [36]. Our study revealed that before enrolment of ALHIV into the psychosocial support intervention, they knew their HIV-positive status without proper counselling, experienced poor psychosocial well-being, mental health, social, emotional, behavioural challenges, and poor treatment literacy. However, ALHIV who were disclosed to (facilitated disclosure) by a trained clinician and recruited into the PSS intervention showed better psychosocial well-being, such as feelings of happiness and not being alone, leading to improved adherence.

A plethora of evidence suggests that health literacy improves medication adherence to and retention in care. A lack of basic HIV knowledge associated with non-adherence was found in a Zambian study [38]. The ALHIV in our study better understood HIV [the virus] and the importance of taking ARV drugs after attending the PSS programme. In this sense, improved treatment literacy about HIV led to improved adherence to and self-management of ART.

We found that the PSS programme facilitated peer-to-peer support, parents/caregivers support, and client–clinician relationships and aided quick access to health service delivery. A study found peer support groups, counselling, supportive healthcare workers, and short waiting times were found to improve adherence to ART [30]. Another study found that family cohesion and social support from caregivers/family were associated with self-reported adherence to ART among HIV-infected adolescents [39]. While one study found the potential of social support interventions to improve mental health [40], another found that a sense of belonging facilitated engagement in ART [41]. Adherence support improved by having a biological mother as a direct supervisor [41].

### Limitations of the Study

This work contains several limitations and opportunities for further research. The current analysis did not investigate how gender identities, age, and duration of attending the support groups may influence experiences of the PSS programme.

In addition, the data saturation technique is mainly employed when analysing and reporting qualitative research based on grounded theory. The study adopted the data saturation technique to strengthen the trustworthiness of the research findings.

## 5. Conclusions

The PSS programme successfully kept ALHIV engaged in ART care despite the health service disruptions encountered during the COVID-19 pandemic. We recommend rigorous evaluation of the effects of the PSS intervention on adherence to and retention in ART among ALHIV in HIV-endemic settings.

## Figures and Tables

**Table 1:** Age and gender breakdown of ALHIV participating in the focus group discussions.

	Females		Males	
Facility Name	10 to 14 Years	15 to 19 Years	10 to 14 Years	15 to 19 Years
Buffelspruit Clinic	9	-	-	-
Kanyamazane CHC	9	12	9	12
Cunningmoore Clinic	-	3	-	3
Langloop CHC	4	4	4	4
Mashinshing CHC	-	-	-	-
Naas CHC	20	30	20	30
Masibeka Clinic	-	-	-	-
Total	42	49	33	49

## Data Availability

The qualitative datasets generated and analysed during the current study are not publicly available but are available from the corresponding author upon reasonable request.

## Appendix A

**Table A1:** Themes, subthemes, and codes by programme objectives.

Category	Theme	Sub Theme	Codes	Quotations		
Pre-intervention						
	Poor psychological well-being	Poor mental health	Depressive symptoms	I cried and did not want to go to school. 15-year-old female, Naas CHC		
			Feelings of hopelessness	I felt like dying as I thought it’s over with my life: I was 9 years when I found out. 17-year-old female, Naas CHC		
		Social challenges	Reclusive	Yes, my parents tried to reach out to me. 14-year-old female, Buffelspruit Clinic		
				I no longer wanted to interact with anyone. 14-year-old female, Buffelspruit Clinic		
				I shut everyone out. 17-year-old female, Cunningmoore Clinic		
			Blaming parents	I was angry at my parents, so I did not want to speak. 17-year-old female, Naas CHC		
		Emotional challenges	Low self-esteem	I became emotional and had low self-esteem. 14-year-old male, Naas CHC		
			Feeling confused	I was confused not knowing what implications that was supposed to have in my day life. 13-year-old male, Cunningmoore Clinic		
			Feeling of anger	I was furious and upset. 15-year-old female, Naas CHC		
			Perceived feeling of lack of affection	Purple, M18: Not even back in the days I was not getting much love before I even knew my status. It’s not nice for me and its painful. 18-year-old male		
		Behavioural challenges	Substance use	I started smoking. 14-year-old female, Naas CHC		
				This changed my life and I started smoking and drinking (laughing) I even started smoking weed so that it would make me high and come back late from friends going home. 16-year-old female, Langloop CHC		
	Poor treatment literacy	Adherence challenges	No awareness of HIV status	Since it was flu medication, I would skip it since I didn’t know that I am to drink for a long time. 15-year-old male, Langloop CHC		
Post intervention		Theme		Sub Theme	Codes	Quotations
		Facilitated disclosure of HIV status		Improved psychological well-being	Feeling “happy”	The white Doctor told me, and I was happy because I finally got an answer of what this medication is for unlike not knowing. 14-year-old female, Langloop CHC
						because on the day they disclosed to me there were other children in the old clinic, and we were told that we are all the same. Meaning we all have the virus and there is nothing to be afraid of, that made me feel happy because there were lots of us that day. 13-year-old female, Langloop CHC
						I am happy because I know my status, unlike not knowing that I am HIV positive. I feel free now because I know my status. 18-year-old female, Naas CHC
					Acceptance of HIV status	My mother told me it does not start with me, and it does not end with me. I should also accept because it is not her fault and as time went by, I started coming to the clinic and I accepted since I am not the only one and I shall overcome it. 18-year-old female, Naas CHC
						As time went by, I accepted because even my dad takes treatment, my uncle as well as my cousin. 19-year-old female, Naas CHC
					Hope for the future	we feel like we have our full life and future ahead of us irrespective of our status. 15-year-old male, Naas CHC
		Health education		Improvement in treatment literacy	Importance of ARVs	I would get sick, and they found out that I was not taking my pills and they reprimanded me for not taking my pills. I was told these pills will help me live and I continued taking them. They also said I should always eat my food so by the time I take my pills I have something in my stomach and that I should drink a lot of water. So, I took that and continued up until now. 13-year-old male
						It is good because it saves our life. 15 year-old male
						….. without your ART you will get sick and lose weight and you will end up dead. 13-year-old female, Langloop CHC
				Better understanding of the virus	HIV knowledge and implications	I think its fine so that the soldiers of the body can stay strong in our bodies. 18-year-old male, Kanyamazane
						It is important to take the medication so that you don’t have AIDS. The treatment makes the body strong and improves the immune system. 13-year-old female
				Improved adherence	Fear of dying motivated continuous uptake	To be honest I was taking my treatment well but when I got to grade 7 that is when the problem began since I was not taking it well. At times I would take it and other times I wouldn’t, so I would have a week without taking it and that happened for a month. When I went back to the clinic for my bloods that was when it was explained to me and my mother that I was not taking my treatment, so they explained more what happens when I do not take it, I will die and from that day, I don’t miss taking my pills. 13-year-old female
					Counselling and encouragement	They encourage and tell us every day that we should take care of ourselves, take treatment on time so that we can be healthy kids and sometimes they take us out for fun walks so that we can relieve stress and that is very helpful to us as kids. 13-year-old female
						because ART would help us on the virus that we have and that will encourage children to take their medication daily. 14-year-old female, Langloop CHC
					Fear of consequences for not taking ARVs	I have accepted and there is someone who was admitted at Rob Fererra hospital two times and the 1st time he was there for 2 months and the 2nd time he was there for 6 months because of not taking treatment. I don’t want to end up like that so I am taking my medication. 18-year-old male
				Self-management of ART	Taking responsibility	No one reminds me about taking ART, I just take them by myself. 14-year-old female
						I am taking responsibility of my health and I remind myself every time and even my little sister does remind me and if I don’t take, she would even report to my mom. So, I thank her a lot she is always there to remind me. 18-year-old female
					Self-reminders using TV programmes	Television also helps, like I know every time generations plays on tv it automatically reminds me to take my ART. 13-year-old male, Langloop CHC
				Understood the seriousness of HIV	Seriousness	It made me realise the seriousness of my status. 15-year-old female
		Strengthened social support network		Peer support	Feeling of not being alone	The support group was good because I felt I was not alone. 17-year-old female
						It is no longer like before and I know that I am not alone and there’s more of us and I can talk to someone about a challenge I have and ask if they have the same challenge and if so we can get to share the solutions and remind each other to take treatment. At least now I do have someone who has experience just like me and I can share with everything I encounter, and I am happy. This support group has really helped me a lot. 18-year-old male
						Sometimes I would feel like that, but I eventually saw one of the kids from school coming to the clinic and attending this group. That is when I saw that I am not alone. 19-year-old female
					Know others taking ARVs	I take my treatment regularly because I am attending with others that are taking treatment. 13-year-old male, Kanyamazane
					Self-esteem	Yes, we need that peer- to-peer support so they can see that they are not the only ones and it’ll be easier for them to talk to us since we’re the same age. We will have confidence and our self-esteem will improve after they assist us. 18-year-old male
						Now I feel proud of myself. I know longer look down on myself. The support I’m getting from this group and home has helped a lot. 13-year-old female, Buffelspruit Clinic
						Because it’s not easy to forget the time they tell you to take your ART. We use the same time to take medication so it’s not easy to forget. 13-year-old female, Langloop CHC
					Share experiences	This group is really helping us as we get share similar experiences about life. 18-year-old male
					Share ideas on how to overcome challenges	We would talk about our medication, like what time others take their ART and other issues relating to our pills. 12-year-old male, Langloop CHC
						I think we should continue with the group because it helps, and we learn from each other, and it becomes easy for us to meet and collect our ART. 13-year-old female, Langloop CHC
						I like the fact that we meet and discuss about similar problems. We are open to each other, and we share similar problems and its nice to know there are others who are going through the same problems as yourself. 13-year-old female, Naas CHC
					Friendship	I love that we communicate and are open with each other. People do not go around gossiping and talking about our status with other people who are not part of this group. 13-year-old male, Naas CHC
						Yes, this group is helping me. I am HIV positive, and I get to sit and listen to other HIV positive peers. 18-year-old male
						I also like this group because I get friends who are also on ART and meet in one place where we help each other. 13-year-old female, Langloop CHC
					Encouraging each other to adherence	We help one another by encouraging each other to take medication every day. 13-year-old female, Langloop CHC
					Coping	Not suffering alone, have comfort and opening to your groups. 14-year-old female, Buffelspruit Clinic
				Parents/caregiver support	Role model	Since both my parents are taking it, that made me to continue taking it because I know that I am not alone
					Family reminds of adherence	She always makes sure that I take my ART regularly, even if she is at work she calls just to check if I have taken my ART. 13-year-old male, Langloop CHC
						My mother reminds me to take ART everyday especially when I forget. 13-year-old female, Langloop CHC
					Emotional support	I used to cry a lot and always wanted to be alone and not talk to anyone. I had a difficult time with my anger, but the person who was very supportive in my life was my grandmother. She helped me with the burden and made me understand that this virus was not a death sentence. 13-year-old female, Buffelspruit Clinic
						I get support from my mom and grandmother, and the rest of the family they don’t know, and even my brothers do not know. 16-year-old female, Cunningmoore Clinic
				Client–clinician relationship	Bonding with clinician	Yes, it does help me because I can ask the nurse a lot of things, and with my peers as well, I can share whatever is bothering me and if I forget to take my pills, I ask them what do they do if they forget or if they don’t. So, it very helpful. 18-year-old female, Naas CHC
					Friendly healthcare worker	She (Professional nurse) is very open and easy to talk to and she is not the type of person who gets angry or even shout at us. She behaves like us so that she can understand us better. 18-year-old female, Naas CHC
				Improved Health service delivery	Quick access to treatment	It’s great to meet in the groups, we get assisted very fast and you arrive home very early because you don’t que. 12-year-old male, Langloop CHC
						So far it is working for me because I don’t take time to que when I am here and here, I just sit for an hour or 30min. 15-year-old female, Kanyamazane CHC
Recommendations to improve PSS						
					Reminders to collect medication	Before, let’s take if I am supposed to come on Sunday, they can call me in the morning and remind me. 13-year-old male, Langloop CHC
					Psychological motivation	We will appreciate if we can have counsellors to motivate us maybe once or twice a year so that we can continue to take our ART with confidence because that encourages and give us hope. 15-year-old female, Buffelspruit Clinic
						If we can get a motivational speaker to motivate us about taking our ART, even if it can be twice a year. 14-year-old female, Naas CHC
					Help with transport to clinic	Maybe help us with transport to reach the facility because we stay far. 14-year-old female, Langloop CHC
					Clinical files of clients treated with confidentiality	Our concern is that when we go to collect our medication, we have files while those who are not on treatment don’t have files so we suggest that a plan be made for us not to be published by files. 17-year-old female, Cunningmoore Clinic
